# Endovascular balloon usage in endoscopic third ventriculostomy for hydrocephalus during a national shortage: case series and technical note

**DOI:** 10.1007/s00381-024-06361-4

**Published:** 2024-04-19

**Authors:** Michael J. Feldman, Hunter Boudreau, Le Tuan Anh, Georges Boubda Tsemo, Jeffrey P. Blount, Curtis J. Rozzelle

**Affiliations:** 1 Division of Pediatric Neurosurgery, Children’s of Alabama, The University of Alabama at Birmingham, Lowder 400, 1600 7th Avenue South, Birmingham, AL 35233 USA; 2 Neurosurgery Department, Vietnam National Hospital of Pediatrics, Hanoi, Vietnam

**Keywords:** Endoscopic third ventriculostomy, Neuroendovascular scepter balloon, Hydrocephalus, Cost, Technical challenges, National shortage, TREK

## Abstract

Endoscopic third ventriculostomy (ETV) is a well-established surgical technique for treating hydrocephalus. Many providers have transitioned to utilizing the specialized Neuroballoon for the stoma dilation in ETV; however, these devices are intermittently unavailable during supply chain shortages. We present the experience of employing cardiac angioplasty and neurovascular balloons as substitutes for the Neuroballoon in 3 patients. The scepter balloon (Microvention), priced at $1800 compared to the standard $300 Neuroballoon (Integra), proved effective, but its pliability presented technical challenges. The substantial cost differential compared to a Neuroballoon ($300) raises economic considerations. The Cardiac TREK balloon (Abbott) was similarly effective, while also being easier to manage endoscopically and cheaper at $158. These experiences support the viability of non-neuroendoscopic specialized balloons as alternatives for ETV dilation of the floor of tuber cinereum.

## Introduction

Endoscopic third ventriculostomy (ETV) is a well-established neurosurgical procedure for the treatment of hydrocephalus [1]. In many centers, balloon dilation is the preferred method of ETV stoma dilation. Traditionally, this technique is performed using the Neuroballoon (Integra) 1. In the setting of a national shortage of standard Neuroballoons, alternative tools have become necessary. This technical note presents our experience with the use of the neuroendovascular Scepter XC balloon as an alternative to the traditional Neuroballoon for stoma dilation during ETV in a single patient.

## Case 1

An 11-year-old male patient with headaches and progressive diplopia required ETV due to hydrocephalus and hyperdynamic aquaductal flow on MRI. Unfortunately, at the time of the procedure, a regional and national shortage of Neuroballoons necessitated the use of an alternative device. The decision was made to proceed with a standard ETV; however, we chose to utilize a neuroendovascular Scepter XC balloon as an alternative.

**Fig. 1:**
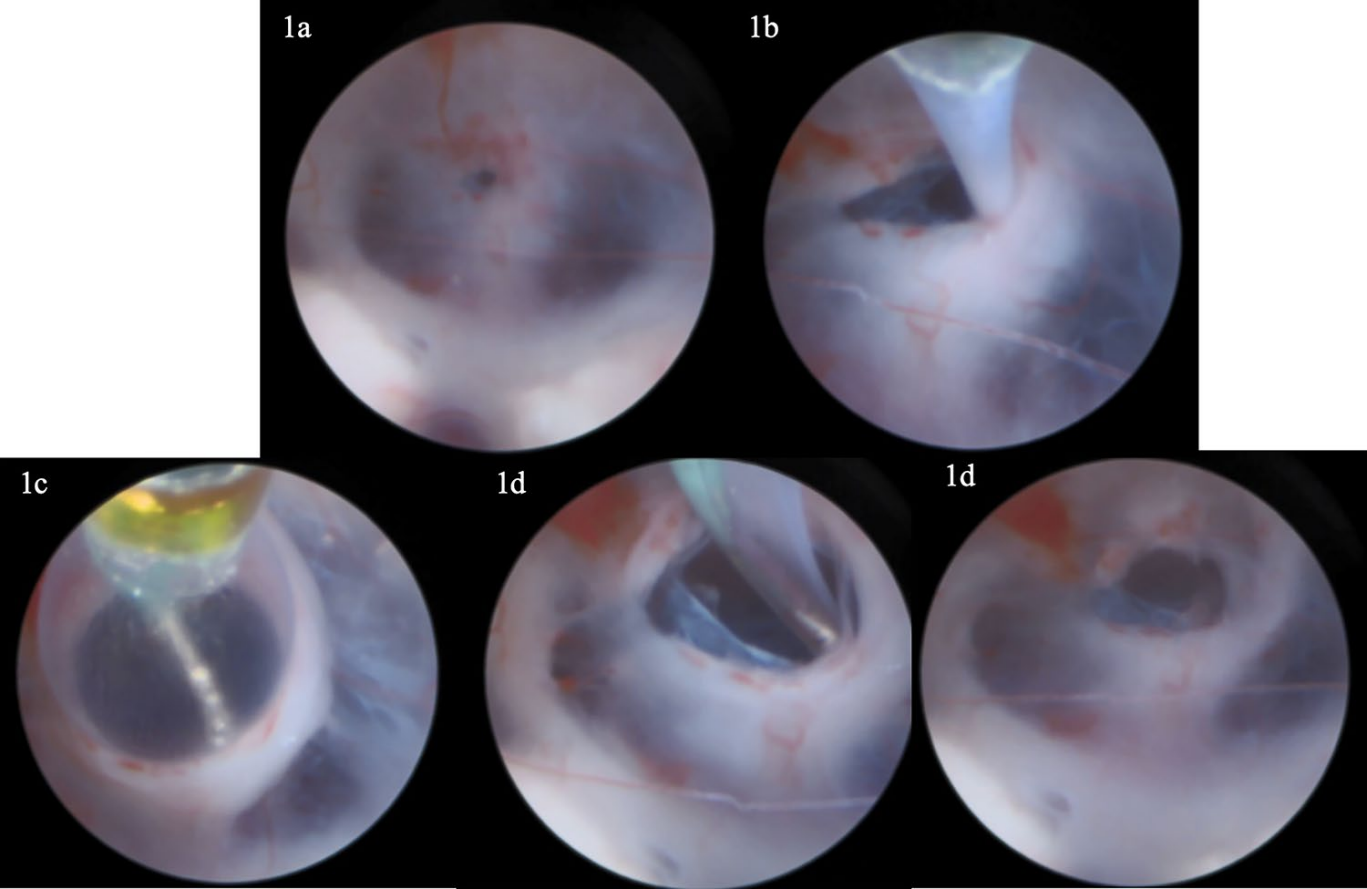
**a** Initial stoma through tuber cinereum. **b** Passing scepter balloon microcatheter through stoma and membrane of Liliequist prior to dilation. **c** Dilation of the balloon microcatheter and stoma expansion. **d** Deflation of the balloon microcatheter and subsequent removal from stoma with demonstration of enlarged stoma

The patient was positioned supine and widely draped. A burr hole was then made abutting the anterior aspect of the coronal suture in the mid-pupillary line. Next, the dura was incised and the ventricle was accessed using a brain needle and a peel-away retractor system. A Little LOTTA (Storz) endoscope (3.6 mm outer diameter) was then introduced to the ventricle and the tuber cinereum was identified. Stoma was made using an endoscopic micropituitary, and the hole in the floor of the third ventricle was noted to extend through the scarred membrane of Lilliequist (Fig. 1a). The Scepter XC balloon, which had been previously prepared on the back table per the manufacturer instructions (save for using normal saline instead of contrast), was then introduced through the working channel of the endoscope and passed under direct visualization through the stoma (Fig. 1b). Once secured in the correct position, the balloon was inflated to the maximum diameter of 6 mm (Fig. 1c), subsequently deflated, and then removed though the endoscope. We immediately noted hyperdynamic movement of the floor of the third ventricle and enlargement of the stoma (Fig. 1d). The endoscope was removed, the wound was closed, and the patient was brought to the recovery room. The patient expressed immediate symptomatic improvement in diplopia and headaches after surgery.

## Case 2

**Fig. 2:**
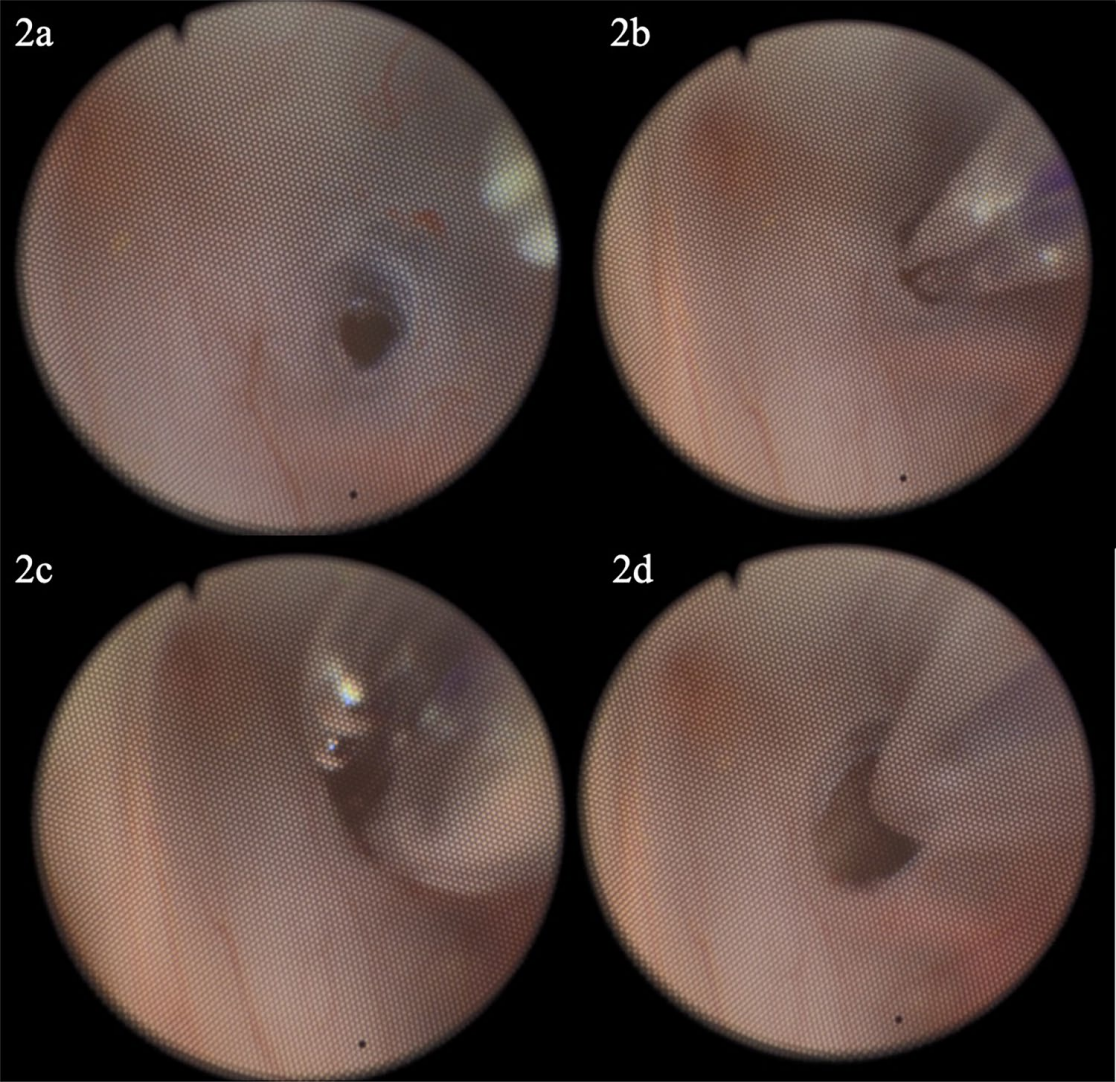
**a** Initial ostomy through floor of third ventricle. **b** Trek cardiac balloon passed through stoma in third ventricular floor prior to dilation with small stoma. **c** Dilation of balloon and expansion of stoma. **d** Deflation of balloon with demonstration of enlarged stoma compared to original in **b**

A 23-month-old female with a history of omphalocele, myelocystocele, and hydrocephalus developed progressive enlargement of her myelocystocele csf space using it as a 5th ventricle. The decision was made to trial ETV as a potential mechanism to improve CSF dynamics. As with the previous case, Neuroballoons were unavailable. Therefore, the decision was made to utilize a 5 × 15-mm TREK monorail balloon (Abbott). Set-up and approach were as above. A burr hole was performed, and the ventricle accessed using a dilator system. A Little LOTTA (Storz) endoscope was introduced, and the stoma performed using a closed endoscopic grasper (Fig. 2a). Next, the TREK balloon which had been prepared per manufacturer instructions on the back table was passed through the working channel, and into the stoma (Fig. 2b). It was then sequentially inflated, deflated, and removed from the stoma (Fig. 2C, D) with significant dilation of the stoma and immediate flow noted through the defect. The wound was then closed, and the patient brought to recovery. There was immediate though moderate improvement noted post-operatively in the patients tense myelocystocele.

## Case 3

**Fig. 3:**
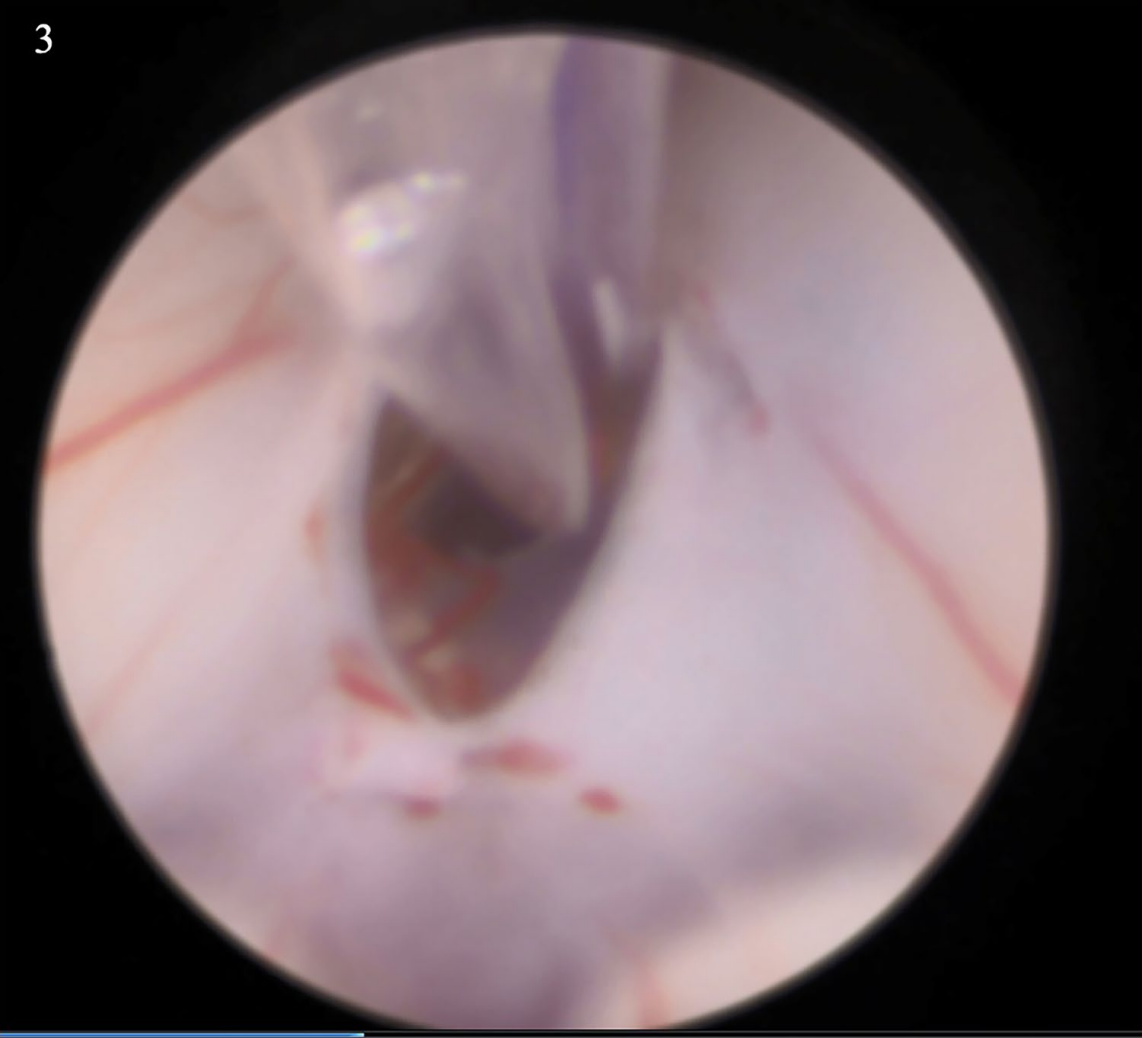
Stoma in the floor of the third ventricle after balloon dilation, with subsequent dissection through the membrane of Liliequist with the tip of the Trek balloon prior to re-inflation

A 4-month-old male with a history of prematurity and progressively growing head circumference, who had originally presented with sunsetting eyes and a tense fontanelle and had an urgent VPS placed, presented to the ED with csf leakage from the abdominal incision. He was taken to the OR for shunt removal and ventriculostomy placement. His cerebrospinal fluid cultures remained negative for several days and he was taken to the OR for ETV with choroid plexus cauterization and ventriculostomy removal. He was positioned supine, and his prior shunt incision opened. Using the Storz flexible endoscope, we entered the ventricle down the previous shunt path, and once there performed the initial stoma with the monopolar device. Next, a TREK 4 × 12-mm balloon was introduced through the working port of the flexible endoscope and passed into the stoma and sequentially inflated/deflated. There were remnant membranes noted in Liliequist membrane, and the tip of the TREK balloon was used to dissect these as well, prior to again inflation then deflation to dilate the perforations within these membranes (Fig. 3). The choroid plexus cauterization was then performed, and the endoscope removed. The patient recovered in the immediate setting well.

## Technical experience

**Fig. 4:**
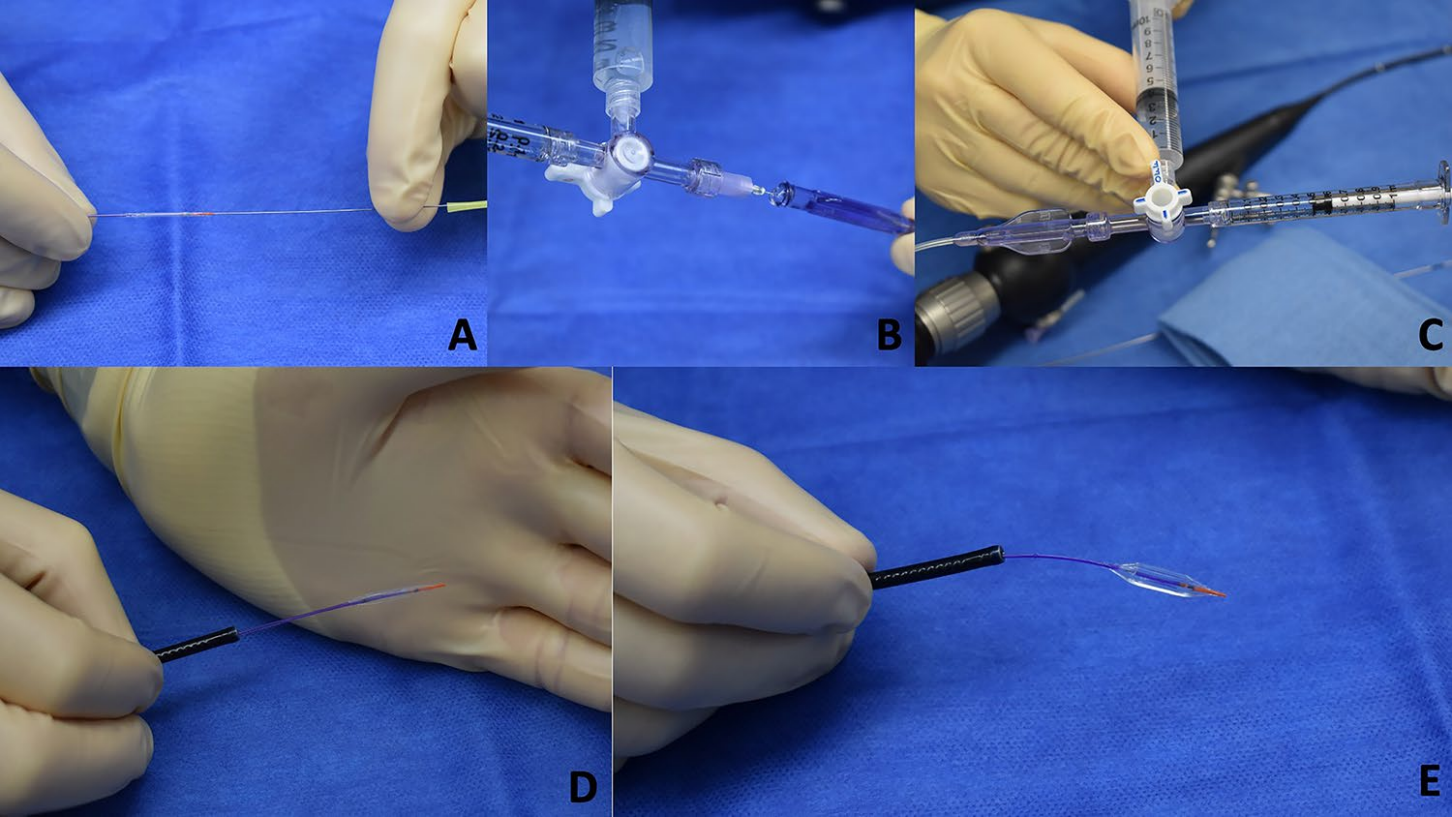
The TREK balloon system is prepared, as per the manufacturer instructions. First, the inner stylet is removed from the deflated balloon **A**. Next, a three-way stopcock is attached to a 1 cc syringe filled with saline, and either a 60 cc aspiration syringe or a 10 cc syringe partially filled with saline. This is used to fill the hub of the catheter with saline and eliminate dead space for inflation **B**. Next, the prepared three-way stopcock is attached to the balloon catheter hub and aspirated from the larger syringe to clear the balloon catheter of bubbles, with the stopcock opened to the smaller syringe once completed **C**. The balloon can then be freely passed through the flexible endoscope in the deflated position **D**. The balloon is easily inflated through the flexible endoscope by flushing with the 1 cc syringe gently, and deflated by aspiration through the larger syringe as adjusted by the three-way stopcock **E**

Although technically and clinically successful, the use of the Scepter XC posed several unique challenges. The device’s flexibility and pliability made it difficult to control during inflation and when navigating within the third ventricle. Specifically, repeated inflation and deflation cycles (as was done to verify inflation prior to case) introduced a gentle bend in the catheter that made navigation through the endoscope somewhat tedious. However, mechanistically, the conformability of the balloon did allow for easy inflation of the balloon within the stoma. Similarly, the TREK balloon performed well in terms of dilation of the defect. Furthermore, its slightly more rigid nature made navigation within the ventricle far easier than the scepter balloon and it was easily passed into the stoma defect from the opening of the fourth ventricular floor. The small non-balloon tip on this device also made a functional dissection tool when the balloon was not inflated. Furthermore, no “watermelon-seeding” or herniation of the balloon cranially or caudally through the stoma was observed, due to the sausage shape of both of these balloons. Moreover, endovascular experience in both preparation and use of the Scepter XC and TREK balloons was of value, as there is a significant learning curve on these devices (Fig. 4).

## Discussion

The use of endovascular devices such as the TREK and Scepter balloon as an alternative to the traditional neuroballoon in this three-patient series provided an effective solution to ETV stoma dilation during a national supply shortage. With the increasingly felt impact of strains on medical supply chains, such uses for alternative devices in surgical settings may become increasingly necessary [2, 3]. Finally, it is important to highlight the significant cost difference between these devices.

The Scepter XC balloon is a dual-lumen micronavigatable catheter designed for distal vessel endovascular navigation that is relatively expensive, costing at $1857.25 at our institution. In comparison, the endoscopic Neuroballoon costs at a fraction of that, at $366.11. Although certainly usable, the significant cost difference between devices and improved ease of use of the Neuroballoon makes it the ideal tool for stoma balloon dilation in normal circumstances. Given our initial patient experience with Scepter, we transitioned in the next two patients to the TREK cardiac angioplasty balloon. This device is slightly stiffer, and significantly less expensive at $158 at our institution. Though not the ideal device, it is certainly a safe and viable alternative when specialized tools are not available.

In the era of just-in-time supply and increasing device-specific shortages, off-label or more expensive devices may more frequently be needed for routine neurosurgical cases. In ETV, this is particularly felt, as there is one formally designed device on-market in the USA for the task of stoma dilation. Alternative methods have described the use of blunt dissection with a probe or the use of a Fogarty balloon; however, these are not always (as at our institution) available in sizes small enough to fit down the working channel of some endoscopes, and are also difficult to position in the stoma during inflation and often “watermelon-seed” out [4]. The direct and indirect costs of these device alternatives do suggest the need for improvement in availability of neurosurgery specific devices, as well as the potential role for development of brand alternatives to commonly used single-use tools.

## Conclusion

In this case series, the Scepter XC and TREK proved to be viable alternatives to the conventional Neuroballoon for ETV stoma dilation during a national Neuroballoon shortage. Evaluation of the cost these devices merits further consideration when deciding on use. Further research and collective experience are needed to fully understand the implications of employing the off-label endovascular balloons as an alternative option during Neuroballoon shortages. However, this experience does demonstrate the important consideration of the impact of supply shortages on patient care, as well as demonstrate viable alternatives for safe performance of ETV.

## Data Availability

No datasets were generated or analysed during the current study.
